# Germination of Seeds and Seedling Growth of *Amaranthus retroflexus* L. Following Sublethal Exposure of Parent Plants to Herbicides

**DOI:** 10.1038/s41598-017-00153-4

**Published:** 2017-03-13

**Authors:** Yue Qi, Bing Yan, Gang Fu, Xiao Guan, Leshan Du, Junsheng Li

**Affiliations:** 1 College of Water Sciences, Beijing Normal University, Beijing, 100875 China; 2 Research Center for Biodiversity, Chinese Research Academy of Environmental Sciences, Beijing, 100012 China

## Abstract

Herbicides have long-term effects on the vegetative parts and reproduction of plants; however, the carry-over effects of herbicides on the F1 generation of invasive plants remain unclear. The objectives of this work were to investigate the germination and growth of the F1 generation of *A. retroflexus*, an invasion plant, treated by sublethal herbicides. The results demonstrated that atrazine or tribenuron-methyl had carry-over effects on the F1 generation of *A. retroflexus*. Atrazine or tribenuron-methyl exposure during the vegetative and reproductive periods significantly inhibited the germination and growth of the F1 generation; a lower sublethal dose of atrazine or tribenuron-methyl did not weaken the inhibition of germination or growth of the F1 generation. Our results suggest that although herbicides have a carry-over inhibition effect on the F1 generation of invasive plants, they may have a more serious carry-over effect on native plants and cause changes in weed species composition and weed diversity.

## Introduction

The development of herbicide-resistant weeds and reductions in non-crop plant richness, abundance and diversity in agro-ecosystems raises concern about the ecological impact of herbicides^1^. When plants are sprayed with herbicides in crop fields and sublethal doses of herbicides reach non-target plants in adjacent habitats through drift, runoff and/or volatilization^2^, the most prominent impact of herbicides is through lethal effects on plants causing changes in plant composition and diversity, or sublethal impact effects causing modification of plant development, growth, and morphology^3^. However the sublethal effects of herbicides on plants are not immediately obvious and may have carry-over effects, which are usually ignored^4^. Although the sublethal effects of herbicide have been given some attention, knowledge of sublethal effects of herbicide on plants remains insufficient^5^.

The sublethal effect of herbicide on the biomass^5, 6^ (including crop yield^7, 8^), reproduction^2, 5, 9^, and physiology^10^ of plants has been reported in several studies, especially research on sublethal effects of herbicide on the biomass and reproduction in greenhouse experiments and field situations^5, 11^. Only a few studies have investigated the carry-over effect of sublethal herbicide on germination and growth of the F1 generation of plants^12, 13^, even though seed germination and the emergence and growth of seedlings are both especially important for plant adaptation and population recruitment as these processes establish the beginning of subsequent plant development and natural selection^14, 15^. There are very few studies on the carry-over effect of sublethal herbicide on the F1 generation of invasive plants, even though germination and seedling growth of invasive plants will affect survival and development^16, 17^, and thus cause changes in weed species composition and weed diversity. In addition, plants sprayed by herbicides during different growth stages, such as the vegetative or reproductive stages have different responses to herbicides^12, 18, 19^. Thus, it is worth investigating whether there are different responses of the F1 generation from parent plants during different growth stages treated with herbicide.

*Amaranthus retroflexus* L. is a common annual C4, monoecious dicotyledonous weed in the Amaranthaceae family^20–22^. The flowers are small, unisexual and develop in numerous dense clusters^23^. It reproduces by seed and produces 5000 to 300,000 seeds per plant^23, 24^. Seedling emergence occurs over several months each year^23, 25^. This species can grow up to 1–2 m and compete with crops for light, nutrients, and moisture, and can reduce crop yields^26–29^. It is one of the world’s worst weeds and is widely distributed in 70 tropical and subtropical countries^23, 30–32^. It is also found in fields and orchards in China^31, 32^. In 2014, it was listed on *the third list of the China exotic invasive species* by the Ministry of Environmental Protection of China and the Chinese Academy of Sciences (http://www.zhb.gov.cn/gkml/hbb/bgg/201408/t20140828_288367.htm).

Here, we aim to study the carry-over effects of sublethal herbicides on the F1 generation of *A. retroflexus* L. as an invasive plant using parent plants in the vegetative or reproductive periods treated by atrazine or tribenuron-methyl, which are two commonly used herbicides in Chinese arable cereal crops^33^ and also commonly used to control *A. retroflexus* in China^34, 35^. Our objectives were: (i) to determine whether there was a carry-over effect of atrazine and tribenuron-methyl on the F1 generation of *A. retroflexus*, (ii) to compare the germination and growth of the F1 generation of *A. retroflexus* treated with herbicides during different growth periods (vegetative and reproductive), and (iii) to determine if an increased sublethal dose to the parent plant increases the toxic effect on the F1 generation.

## Methods

### Site description

Seeds of *A. retroflexus* were collected for an outdoor pot experiment at the experiment station of Chinese Research Academy of Environment Science. The location was the town of Zhaoquanying, Shunyi District, Beijing, China (115.7°-117.4°E, 39.4°-41.6°N; 20–60 masl). Beijing has a semi-humid monsoonal climate with distinct seasons. The mean temperature is −4 °C in January and 26 °C in July and August. The annual surface evaporation is 1800 mm, and the mean annual rainfall is 655 mm. The precipitation is unevenly distributed, with more than 80% occurring during June, July, and August^36^.

### Test herbicides

Atrazine (2-Chloro-4-ethylamino-6-isopropylamino-1,3,5-triazine) (GREEN LAND® Shandongshengbang greenland Chemical Co., Ltd) binds to the plastoquinone binding site (Qb) in photosynthetic electron transport and halts photosynthesis^37, 38^. This chemical is in the triazine herbicide class and is used as a soil and leaf treatment herbicide. The recommended application rate of atrazine in North China is 1200 g ai/ha. Tribenuron-methyl (methyl 2-[4-methoxy-6-methyl-1,3,5-triazin-2-yl(methyl)carbamoylsulfamoyl]benzoate) (QCC® Shandong Qiaochang Chemical Co., Ltd) inhibits acetolactate synthase (ALS), which is a key enzyme in the biosynthesis of branched-chain amino acids. It is rapidly absorbed by plant leaves^39^. The recommended application rate of tribenuron-methyl in North China is 22.5 g ai/ha.

### Plant material and culture condition

On May 6, 2014, seeds of *A. retroflexus*, collected from an untreated herbicide population in abandoned farmland of the experimental station, were sown in 174 plastic pots outdoors at a rate of approximately 10 seeds per pot. The potting soil used was Fluvo-aquic. After seedlings had developed 2–3 true leaves, they were thinned to one per pot. Seedlings were watered every day. All plants were supplemented with 50 mL of a prepared solution consisting of 2.5 mL/L of 20-20-20 “All Purpose Plant Food fertilizer” (America chemcore biochemistry technology group CO., LTD.) at approximately 30 d and 60 d after seedlings emerged^40^. Six seedlings were exposed to one of the following atrazine doses: 1200 g ai/ha (the recommended field application concentration, RFAC), 600 g ai/ha (1/2 of RFAC), 300 g ai/ha (1/4 of RFAC), 150 g ai/ha (1/8 of RFAC), 75 g ai/ha (1/16 of RFAC), 37.5 g ai/ha (1/32 of RFAC), 18.75 g ai/ha (1/64 of RFAC), or tribenuron-methyl doses: 22.5 g ai/ha (RFAC), 11.25 g ai/ha (1/2 of RFAC), 5.63 g ai/ha (1/4 of RFAC), 2.81 g ai/ha (1/8 of RFAC), 1.41 g ai/ha (1/16 of RFAC), 0.70 g ai/ha (1/32 of RFAC), 0.35 g ai/ha (1/64 of RFAC). A total of 84 seedlings with 12 to 14 true leaves (TL) were sprayed with herbicide using a manual sprayer with cone-shape spray nozzles (worth® NS-5, China), and another 84 seedlings in the early blooming (EB) period were treated only on the spikes (length < 5 cm) using a paintbrush^41^. Six non-treated were used as blank controls. No additional surfactants or other adjuvants were used in the treatments. The six replicate plants treated with same herbicide dose were isolated from those treated with different doses for 48 h after herbicide application. At 48 h, the plant locations were randomized.

Seeds of *A. retroflexus* were collected as they matured. The seeds were cleaned to remove the husk and other debris and were maintained dry in a ventilated room. The seeds from six plants treated with the same doses of herbicide were mixed and stored in one opaque paper bag at a constant temperature (4 ± 0.5 °C) for three months until the start of the germination tests^42^.

### Seed germination test

Seeds of similar size were placed in standard petri dishes (90-mm diameter) on two pieces of filter papers (90-mm diameter) moistened with 4 mL of distilled water. Four replicates of 50 seeds from plants treated with the same dose of herbicide were germinated under constant conditions: 25 °C and 12-h light/12-h dark with a relative humidity of 65% for 28 days^20, 43, 44^. Germinated seeds from the petri dishes were counted and placed in similar petri dishes without a cover every day at 24-h intervals. Seeds were considered germinated once the radical was 1–2 mm long^12, 45^. A total of 5–12 seeds were selected randomly from each replicate for the length measurement on the 7^th^ day from the start of the germination experiment. The radicle-hypocotyl junction was identified by the color of the germination shoot (the white colored part was the radicle, the red part was the hypocotyl)^45^.

Radicle length, hypocotyl length of 7-day-old seedlings were measured, and percent germination^46^, mean germination time, initial germination time of seeds^45, 47^, total length of radicle and hypocotyl, and ratio of radicle length to hypocotyl length of 7-day-old seedlings were calculated.

### Statistical analysis

All data were analyzed for the main effects of different growth stages of plants treated with herbicide, herbicide type, herbicide dose, and their interaction using MANOVA. One-way ANOVA or independent-sample t test were analyzed for the main effects. After carrying out one-way ANOVA, the Fisher’s Protected LSD test was used to detect significant differences (*P *< 0.05) among the treatments. Data were analyzed using the non-parametric Kruskal-Wallis test if transformational data did not satisfy the assumption of homogeneity of variance. The Kruskal-Wallis test was followed by all pairwise multiple comparisons. MANOVA, ANOVA and independent-sample t test were employed to test the differences between means from the experiments. Data are shown as the mean ± standard deviation (SD).

## Results

### Carry-over effects of herbicides

**Figure 1:**
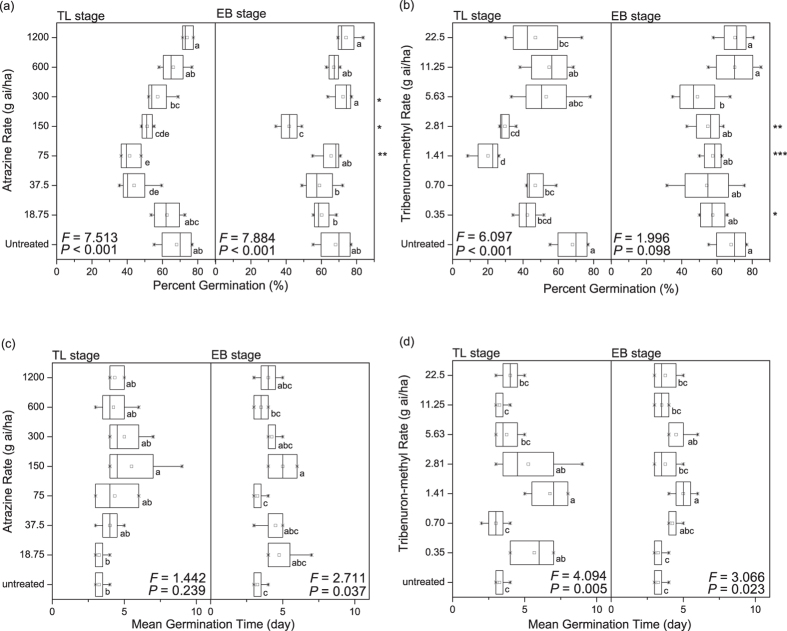
Percent germination (**a,b**) and mean germination time (**c,d**) of seeds produced after the 12 to 14 true leaves stage (TL stage) and the early blooming stage (EB stage) treated with atrazine (**a,c**) or tribenuron-methyl (**b,d**). In the boxplots here, the ends of the box represent the 25^th^ and 75^th^ percentiles; the bars inside the box represent the 50^th^ percentile, or the median, and the ends of the whiskers represent the minimum and maximum values. Means are represented by solid circles. Different letters indicate significant difference between different doses of the same herbicide at α = 0. 05. *Shows the influence of different plant growth stages treated with the same herbicide dose according to the independent-sample t test. *P < 0.05, **P < 0.01.

**Figure 2:**
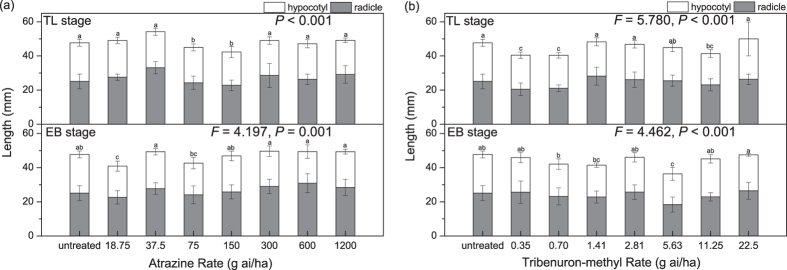
Length of radicle and hypocotyl of 7-day-old seedlings produced after the 12 to 14 true leaves stage (TL stage) and the early blooming stage (EB stage) treated with atrazine (**a**) or tribenuron-methyl (**b**). Here, and in Fig. 3, different letters indicate significant difference between different doses of the same herbicide at α = 0. 05; and the Kruskal-Wallis test results had no *F* values.

*A. retroflexus* treated with atrazine or tribenuron-methyl inhibited percent germination (Fig. 1a,b) and radicle and hypocotyl growth (Fig. 2a,b), and delayed mean germination time of the F1 generation (Fig. 1c,d). However, neither herbicide affected initial germination time. Initial germination of the seeds generally started on the second or third day of the germination experiment.

**Figure 3:**
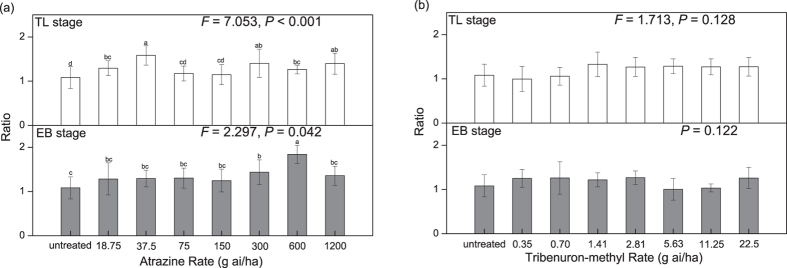
Ratio of radicle length to hypocotyl length of 7-day-old seedlings produced after the 12 to 14 true leaves stage (TL stage) and the early blooming stage (EB stage) treated with atrazine (**a**) or tribenuron-methyl (**b**).

On the other hand, compared to atrazine, tribenuron-methyl had greater inhibition of percent germination of the F1 generation (Fig. 1a,b). Atrazine increased ratio of radicle length to hypocotyl length (Fig. 3a), while tribenuron-methyl had no significant effect on the ratio (Fig. 3b). Herbicide type had significant effect on percent germination, radicle length, length of radicle and hypocotyl, and ratio of radicle length to hypocotyl length of the F1 *A. retroflexus* (Table 1).

**Table 1:** MANOVA results (*F* values) for testing for different growth stages of parent plants treated by herbicide, herbicide type, dose and their interactions on germination and 7-day-old seedlings growth of *A. retroflexus* F1 generation.

Source of variation	d.f.	GP	MGT	IGT	HL	RL	LRH	RRH
Herbicide type (HT)	1	20.402***	0.006	0.576	0.303	37.223***	25.233***	22.034***
Growth stage (GS)	1	32.392***	2.654	1.599	1.493	1.433	1.357	0.422
Herbicide dose (HD)	7	12.927***	4.180***	0.804	5.576***	2.702*	3.636**	4.289***
HT × GS	1	7.088**	0.520	0.064	0.278	0.120	0.076	2.096
GS × HD	7	3.138**	1.563	1.181	1.035	2.267**	2.062*	1.278
HT × GS × HD	7	3.050**	2.291*	1.105	3.276**	5.989***	4.783***	5.712***

GP = germination percent; MGT = mean germination time; IGT = initial germination time; HL = hypocotyl length; RL = radicle length; LRH = the length of radicle and hypocotyl; RRH = the ratio of radicle length to hypocotyl length. d.f.: degrees of freedom; ns: not significant; *P < 0.05; **P < 0.01; ***P < 0.001.

### Effects of herbicides used at different growth stages

The herbicide applied during the 12 to 14 true leaves stage (TL stage) or the early blooming stage (EB stage) of *A. retroflexus* inhibited percent germination (Fig. 1a,b) and radicle and hypocotyl growth (Fig. 3a,b), and increased mean germination time of the F1 generation (Fig. 1c,d). However, inhibition of percent germination by applying herbicide during the TL stage was higher than that during the EB stage of *A. retroflexus*; notably, percent germination of seeds from parent plants treated with 1/16 of the recommended field application concentration (RFAC) of herbicide (75 g ai/ha of atrazine or 1.41 g ai/ha of tribenuron-methyl) during the TL stage was significantly lower than during the EB stage (*P *< 0.01, atrazine; *P *< 0.001, tribenuron-methyl) (Fig. 1a,b). Different growth stages with the herbicide had significant effect on percent germination, but had no significant effect on mean germination time, initial germination time, hypocotyl length, radicle length, length of radicle and hypocotyl, and ratio of radicle length to hypocotyl length of the F1 *A. retroflexus* (Table 1).

### Effects of herbicide dose

Herbicide dose had significant effect on percent germination, mean germination time, hypocotyl length, radicle length, length of radicle and hypocotyl, and ratio of radicle length to hypocotyl length of the F1 *A*. *retroflexus* (Table 1). The inhibition of seed germination and seedling growth did not increase as herbicide dose increased. With the increase of herbicide dose, percent germination decreased and then rebounded, which showed a “V” shape, and the lowest point was the percent germination of seeds from parent plants treated with 1/16 of RFAC of herbicide (75 g ai/ha of atrazine or 1.41 g ai/ha of tribenuron-methyl, TL stage) (Fig. 1a,b). Moreover, with the increase of herbicide dose, mean germination time of seeds was prolonged and then shortened, which showed a reverse “V” shape, and the inflexion point was the mean germination time of seeds from parent plants treated with 1/8 of RFAC of atrazine (150 g ai/ha) or 1/16 of RFAC of tribenuron-methyl (1.41 g ai/ha) (Fig. 1c,d). Compared with RFAC of atrazine or tribenuron-methyl, the lower doses of the herbicide had a greater influence on germination and seedling growth.

### Interactions

Herbicide type, herbicide dose and growth stage (herbicide applied during different growth stage of parent plants) had interactions on germination and seedling growth of the F1 *A*. *retroflexus.* The interactions between herbicide type and growth stage only significantly impact percent germination; and the interactions between growth stage and herbicide dose significantly impact percent germination, radicle length, and total length of radicle and hypocotyl (Table 1). Such as tribenuron-methyl applied during the TL stage or the EB stage inhibited percent germination of the F1 *A. retroflexus*, but the lowest percent germination of seeds was found in parent plants during TL stage with 1.41 g ai/ha, whereas EB stage with 5.63 g ai/ha of tribenuron-methyl. The interactions between herbicide type, growth stage and dose significantly impacted all observation endpoints except initial germination time (Table 1).

In addition, initial germination time of the F1 *A. retroflexus*, which may be a non-sensitive parameter, was not significantly affected by herbicide type, growth stage, herbicide dose, and the interactions; while percent germination, which may be a sensitive parameter, was significantly affected by all these factors (Table 1).

## Discussion

Herbicide application^48, 49^ and invasive plants^50^ are both causes of the decline in native species diversity and changing composition of native communities in agricultural landscapes. Invasive plants as arable weeds have to withstand high dose of herbicides applied in crop fields, as well as sublethal dose drift from crop fields along field margins and boundaries. Several studies have found that long-term effects of herbicides on flowering, seed production and the F1 germination of many weeds^1, 2, 12, 51, 52^, as well as our data on invasive plants, supports the above view. In this experiment, herbicide application, especially the lower sublethal dose, had a significant long-term influence on the germination and growth of the F1 generation of *A*. *retroflexus*. The long-term effect will influence the population development of this plant as an annual species, which relies completely on seed propagation; however, it is difficult to determine whether the F1 generation and the population had a lower competitive advantage within the community. The germination of *A*. *retroflexus* seed increased after 1 yr of burial^25^; consequently, a low germination rate of the F1 generation of *A*. *retroflexus* may lead to the maximum supplementation of their soil seed bank to ensure their population persistence. Furthermore, although seed behavior traits (dormancy, germination rate and seed ageing) are not directly linked to the current herbicide resistance level^53^, plants treated with low rate of herbicides result in a rapid evolution of herbicide resistance^54^. Seedlings of *A*. *retroflexus* from parent plants exposed to sublethal herbicides may result in a more serious threat to agricultural production and native plant diversity.

Contrary to our assumptions, overall, the herbicide inhibition effect on the germination and growth of the F1 generation did not increase as the sublethal dose increased, similar to other studies^13^. Our data on the germination and growth of the F1 generation affected by different sublethal doses showed a “V” shape change trend. A trend similar to a V may be explained by plant resistance. The herbicides had no carry-over effect on the F1 generation at the lower dose; as the herbicide dose increased, the toxicological pressure on the F1 generation increased; as the herbicide dose continued to increase, the physiological resistance of *A*. *retroflexus* may have reduced the toxic effects on the F1 generation. From another perspective, we could explain the inflexion point of a V-shape change trend by *survivorship bias* ^55^. As a sublethal dose of herbicide continues to increase, reproduction organs may be not able to survive before growing into mature seeds, e.g. bud abortion and unfilled seed^52^; thus, surviving seeds which could grow into mature seeds under herbicide spray, had strong vitality that may result in high germination and rapid growth. We identified that the lower sublethal dose of atrazine or tribenuron-methyl, compared with the higher sublethal dose, did not weakly inhibit F1 generation germination and growth of *A*. *retroflexus*, while in other cases, the inhibition of tribenuron-methyl on F1 generation germination of *Fallopia convolvulus* and *Galiumspurium* increased as the sublethal dose increased^12^. In addition to the herbicide chemical properties and different responses of plant species^3^, the herbicide dose is one of the key factors that influences plants^40^. Thus, the different influence of herbicide doses, especially the sublethal doses, should not be neglected, otherwise the ecological risk of herbicide sublethal doses that drift from crop fields may be underestimated.

Our data showed that, as expected, herbicide applied during the vegetative and reproductive stages of *A*. *retroflexus* had carry-over effects on the F1 generation, similar to other studies on wild plants and crops^12, 19, 52, 56, 57^. And different growth stages treated with herbicides had different influences on the F1 generation of *A*. *retroflexus*, e.g. the percent germination. The interaction between growth stage and dose had a significant effect on seedling growth. To summarize, herbicide application time is an important influencing factor on the F1 generation of *A*. *retroflexus*. This illustrates that the vegetative and reproductive stages of *A*. *retroflexus* resulted in different sensitivity to different doses of herbicide.

Herbicide effectiveness, resulting from differences in the application timing, may be related to the direct damage caused to different plant organs. The use of herbicides during the vegetative stage of plants could harm the stems and leaves. Leaves are plant food producing organs, so influencing the assimilate production by herbicidal action will affect the storage and allocation of photosynthate and reduce plant growth and reproduction^4, 37, 40^. For some plant species treated with herbicides, biomass recovery was not accompanied by comparable levels of reproductive recovery, and the energy consumed during the biomass recovery period may reduce reproduction^4^. Additionally, herbicides could directly damage plant reproductive organs or the reproductive process^52, 58^, and our results support the above view. We used different organs (entire plant in the 12 to 14 true leaves period and inflorescence in the early blooming period) of *A*. *retroflexus* treated with herbicides at different growth stages as the targets of the herbicide application; entire plant as the targets of the herbicide application have been adopted in many previous studies^18, 19, 56, 57, 59, 60^. Greater effects were observed on seeds when herbicide was applied during early reproductive rather than later growth stages because embryo cell division is rapid for a time following fertilization but then subsequently slows^19^.

These findings must be interpreted cautiously for several reasons. Our experiments only tested one invasive plant; therefore, they represent just one sample of the Chinese exotic invasive species, and two commonly used herbicides from a long list of herbicides that are frequently applied in Chinese farmland^61–63^. Moreover, we only tested the germination and growth of the F1 generation; previous reports showed that herbicide has a carry-over effect on the dormancy of the progeny^53^. Furthermore, we did not consider the relationship between invasive plants and native plants, which may result in an underestimate of the ecological risk arising from a sublethal dose of herbicide applied to invasive plants. Thus, further studies on the ecological risk of carry-over effects of herbicides on invasive plants should consider more invasive plants, and in particular, compare the response of invasive plants to that of native plants, using different herbicides that are typically applied in Chinese farmland, as well as test the germination and dormancy of the F1 generation.
